# Real-world insights on preferred treatments for steroid-refractory immune checkpoint inhibitor-induced pneumonitis

**DOI:** 10.1136/jitc-2020-002252

**Published:** 2021-02-18

**Authors:** Margaret Gatti-Mays, James L Gulley

**Affiliations:** 1 Pelotonia Institute for Immuno-Oncology, Division of Medical Oncology, The Ohio State University Comprehensive Cancer Center, Columbus, OH, USA; 2 Genitourinary Malignancy Branch, National Cancer Institute, Bethesda, Maryland, USA

**Keywords:** immunotherapy, programmed cell death 1 receptor, guidelines as topic

Immune checkpoint inhibitor (ICI)-induced pneumonitis has been considered a rare immune-related adverse event (irAE). Incidence is estimated at less than 5% of clinical trial patients, with high-grade pneumonitis occurring in less than 1% of patients who receive ICIs.^1 2^ However, as ICIs gain traction as standard therapies, real-world data suggest a slightly higher incidence than is seen in clinical trial populations, with ICI-induced pneumonitis occurring in up to 19% of patients. The risk of developing ICI-induced pneumonitis varies among ICI classes. Anti-programmed cell death 1 (PD-1) inhibitors have a higher incidence than anti-PD-L1 inhibitors or anti-cytotoxic T-lymphocyte-associated protein 4 (CTLA-4) inhibitors, and combination therapy results in a higher risk of pneumonitis compared with ICI monotherapy.^3^ In addition, ICI-induced pneumonitis is most common in patients with lung cancer and is the most common anti-PD-1/L1-related cause of death across all tumor types.^2 4^

ICI-induced pneumonitis generally develops within the first 3 months of treatment.^5^ High-dose steroids such as prednisone/methylprednisolone 1–2 mg/kg/day are used initially. If patients do not improve after 48–72 hours on high-dose steroids, they are classified as steroid refractory. Current consensus guidelines by National Comprehensive Cancer Network (NCCN),^6^ Society for Immunotherapy of Cancer,^1^ and European Society for Medical Oncology (ESMO)^7^ recommend escalating immunosuppression with the addition of one of the following agents to high-dose steroids for steroid-refractory irAEs: (1) infliximab 5 mg/kg, (2) cyclosporine 2.5 mg/kg/day in divided doses, (3) mycophenolate mofetil 1–1.5 g two times a day, (4) intravenous immunoglobulin (IVIG) 2 g/kg in divided doses over 2–5 days, or (5) tocilizumab 0.6 mg/kg. However, consensus guidelines do not specify the preferred initial agent, the optimal order of agents, or the optimal number of doses. The tumor necrosis factor (TNF)-α inhibitor infliximab is often the preferred first option as it has been used successfully in other irAEs such as colitis, but there are little published data on its efficacy in treating steroid-refractory ICI-induced pneumonitis.^8^

Balaji *et al*^9^ and Beattie *et al*^10^ present the largest retrospective chart reviews to date (n=12 and 26 patients, respectively) on the clinical management and course of patients at their respective institutions with steroid-refractory ICI-induced pneumonitis. Given the low incidence of pneumonitis and the even lower incidence of steroid-refractory pneumonitis, these retrospective reviews provide an important clinical insight into the efficacy of recommended therapies (specifically infliximab) and long-term clinical outcomes of patients with steroid-refractory ICI-induced pneumonitis.

In both studies, most patients who developed ICI-induced pneumonitis had lung cancer, which is consistent with prior studies.^2^ It is not surprising that at least 75% of patients were current or former smokers and many had prior chest radiation. However, only a small number had clinically evident pre-existing pulmonary conditions. Hypotheses about increased incidence of pneumonitis in patients with lung cancer who receive ICI include greater tumor burden in the lungs than other cancers, pre-existing lung conditions, and prior pulmonary damage from tobacco use. While this causal association has previously been documented, it is important to note that tobacco-associated lung cancers generally have a higher tumor mutational burden, higher PD-L1 expression and respond better to ICIs. The retrospective studies presented here raise important questions about the true risk of current or prior tobacco use and the development of pneumonitis. This causal association has yet to be verified in prospective studies. Additional studies needed to better identify which patients are at higher risk of developing pneumonitis, specifically primary steroid-refractory ICI-induced pneumonitis.

Balaji *et al* describe the clinical management and disease course of 12 patients with primary steroid-refractory ICI-induced pneumonitis (defined by the authors as lack of clinical improvement after at least 48 hours and up to 14 days of high-dose corticosteroids). The identified patients were referred to the multidisciplinary immune-related toxicity team or pulmonary outpatient clinic with ICI-induced pneumonitis. In this study, primary steroid refractoriness occurred after a median of 5 doses of ICI, resulting in grade 2 (n=3) and grade 3 (n=9) symptomatic pneumonitis. The most commonly identified radiographic pattern was diffuse alveolar damage. The mean dose of steroids administered was prednisone equivalent 75 mg/day prior to receiving additional immunosuppressive therapy. Clinical management included treatment with IVIG (n=7; started a mean of 17 days after steroid initiation), infliximab (n=2; started 7 and 8 days after steroid initiation), and combination IVIG + infliximab (n=3; infliximab added to IVIG on days 8, 9, and 15). Almost all patients with primary steroid-refractory ICI-induced pneumonitis required management in the intensive care unit; around half required mechanical ventilation. The authors report that 75% of patients in this study died of steroid-refractory ICI-induced pneumonitis or infectious complications, including all five patients treated with infliximab (two as monotherapy, three in combination with IVIG).

Beattie *et al* describe the clinical course of 26 patients who had either primary steroid-refractory ICI-induced pneumonitis (n=12; defined as no improvement or worsening of pneumonitis with initial high-dose steroid monotherapy) or steroid-resistant ICI-induced pneumonitis (n=14; defined as initial response to steroid with subsequent recurrence of pneumonitis during steroid tapering in the absence of ICI rechallenge). The authors searched for administration of immunomodulatory agents and evaluated the clinical management and course for patients who clinically and/or pathologically had ICI-induced pneumonitis. All patients with primary steroid-refractory ICI-induced pneumonitis were treated with at least 60 mg of prednisone equivalent. Median time to addition of an immunomodulator for primary steroid-refractory ICI-induced pneumonitis was 7 days, and median time to additional treatment for steroid-resistant pneumonitis during steroid taper was 2.9 months. The study reported more severe disease in patients with primary steroid-refractory ICI-induced pneumonitis (grade 2 = 1, grade 3, n=9; grade 4, n=2) than in patients with resistant pneumonitis (grade 2, n=10; grade 3, n=4; grade 4, n=0). Most of the retrospective study included patients treated with infliximab monotherapy (n=15; 58%), mycophenolate mofetil monotherapy (n=5; 19%) or infliximab comination therapy (n= 5; 19%). However, further investigation into the subgroups shows that all patients with primary steroid-refractory pneumonitis (n=12) received infliximab as monotherapy or in combination with mycophenolate, and most patients with recurrent pneumonitis received infliximab, and/or mycophenolate. Of the 26 patients identified, more than half (62%) were hospitalized and, despite aggressive treatment, only 10 had a durable improvement following additional immunosuppression plus steroids. However, when focusing on the primary steroid-refractory patients, only 3/12 (25%) had durable desponses to combination immunosuppression and all 12 patients died with cause of death attritbued to pneumonitis or infectious complications in half of these patients (See supplemental table 3).

Steroid-refractory ICI-induced pnemonitis is a rare irAE with a poor prognosis. Patients with primary steroid-refractory ICI-induced pneumonitis have a high all-cause mortality rate (pneumonitis or infectious complications) and a worse prognosis than patients with recurrent ICI-induced pneumonitis. Identifying ICI-induced pneumonitis may be challenging due to varying clinical presentations, but the most common radiographic pattern is diffuse aveolar damage. It is unclear if rapid escalation from steroid monotherapy to combination therapy with other immune modulators can fully reverse the disease course or improve clinical outcomes in patients with high-grade primary steroid-refractory ICI-induced pneumonitis. However, therapy escalation may lead to durable improvement (although rarely complete resolution) in steroid-refractory ICI-induced pneumonitis in up to 30%–40% of patients. Prospective studies on the efficacy of immune modulators or combinations are greatly needed for primary steroid-refractory ICI-induced pneumonitis. Furthermore, in addition to standard endpoints like 90-day morbidity and duration of immunosuppression, these trials should also incorporate clinically meaningful endpoints like FEV1, required level of clinical care, and need for supplemental oxygen. Prospective studies should also evaluate the role of patient-specific factors like current or prior tobacco use and prior radiation on the development of irAEs. Finally prospective studies should aim to incorporate exploratory analyses of peripheral blood, bronchoscopy specimens and tumor samples to investigate potential predictive biomarkers that may indicate an increased risk for the development of pneumonitis. Several prospectitve studies evaluating alternative treatments for steroid-refractory irAEs have recently opened (NCT04438382; NCT04375228; NCT04552704). We eagerly await the results.

Infliximab is a commonly used immune modulator in patients with primary steroid-refracory ICI-induced irAEs, including ICI-induced pneumonitis. These retrospective studies, the largest published to date, demonstrated a high mortality rate (due either to progressive pneumonitis or infectious complications from infliximab) in patients with primary steroid-refractory ICI-induced pneumonitis who received infliximab. While patients with primary steroid-refractory ICI-induced pneumonitis have more severe disease than patients with recurrent ICI-induced pneumonitis, these studies raise serious questions about the efficacy of infliximab in treating steroid-refractory, ICI-induced pneumonitis. Of the 17 patients with primary steroid-refractory ICI-induced pneumonitis who received infliximab with high-dose steroids and/or another immunomodulatory agent (Balaji *et al*=0/5; Beattie *et al*=3/12), only 3 (18%) showed durable clinical responses and all had died. This low treatment response rate with infliximab in primary steroid-refractory ICI-induced pneumonitis demonstrates the need for academically driven, prospective research on treatment algorithms for irAEs. Given that Balaji *et al* demonstrated that patients who received IVIG monotherapy had improved oxygen requirements, a reduced level of care, and reduced ICI-induced pneumonitis-related fatalies, IVIG should be considered as a reasonable alternative to infliximab until prospective irAE data are generated.
